# Hepatic T cell lymphoma after chimeric antigen receptor T cell therapy for myeloma

**DOI:** 10.1002/jha2.1028

**Published:** 2024-10-10

**Authors:** Joshua Mehr, Mingyi Chen

**Affiliations:** ^1^ Department of Pathology University of Texas Southwestern Medical Center Dallas Texas USA

**Keywords:** Chimeric antigen receptor T‐cell therapy, T‐cell lymphoma

1

A 56‐year‐old female with refractory/relapsed multiple myeloma (MM) status post autologous stem cell transplant (6 years ago) and chimeric antigen receptor (CAR) T‐cell therapy (D0 30 days ago) presented with multiple liver nodules. Eight years prior, the patient was diagnosed with immunoglobulin G Lambda MM with 30% involvement in the bone marrow and hyperdiploidy, monosomy 13 cytogenetics. She responded to initial chemotherapy with decreased serum M protein, but her disease continued. Post‐transplant, her disease relapsed with progressively increasing M spike and marrow involvement. After reinduction, pre‐CAR T bone marrow biopsy was negative for minimal residual disease. After fludarabine/cyclophosphamide conditioning, she received Arcellx ddBCMA CAR‐T infusion. She developed grade 2 cytokine release syndrome and was given tocilizumab/dexamethasone. Serology studies for Cytomegalovirus and Epstein‐Barr Virus testing were negative.

Post‐CAR‐T therapy, she developed worsening, headache, nausea, vomiting, and blurred vision which prompted the hospital visit. Laboratory work‐up revealed markedly increased liver enzymes (> 30x upper limit). Imaging showed many rapidly developing liver nodular lesions. A liver biopsy revealed a dense lymphoid infiltrate with variable‐sized atypical lymphocytes infiltrating the hepatic parenchyma. By immunohistochemistry, the abnormal T‐cells were diffusely positive for CD2, CD3, CD4, and CD5 (partial) and negative for bcl2, CD7, CD8, TIA‐1, granzyme‐B, CD30, CD56, PD1, and Epstein‐Barr virus‐In situ hybridization (Figure 1). T cell receptor‐gamma gene rearrangement for T‐cell clonality was positive. No CAR transgene was detected by quantitative polymerase chain reaction. Findings were consistent with peripheral T‐cell lymphoma, not otherwise specified. The patient was treated with dexamethasone (10 mg IV, three doses) which resulted in normalization of liver enzymes and significant reduction of the liver nodules.

**FIGURE 1:**
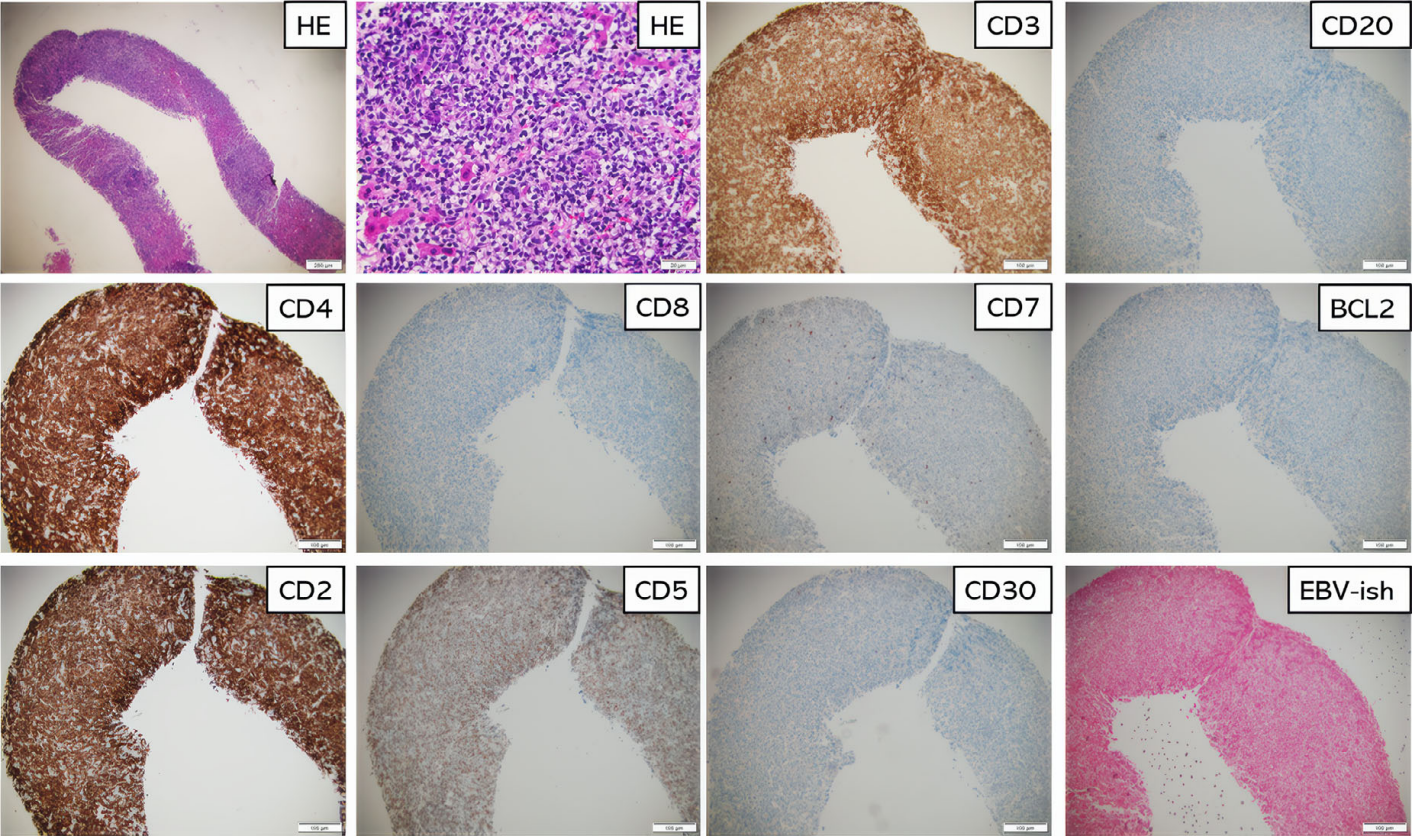
Legend: First Row (Left to Right): Microscopic images showing hepatic T‐cell infiltrate (X100) and (X200), CD3 positivity (X100), CD20 negative (X100). Second Row (Left to Right): CD4 positivity (X100), CD8 negative (X100), CD7 negative (X100) and BCL2 negative (X100). Third Row (Left to Right): CD2 positivity (X100), CD5 partial positivity (X100), CD30 negative (X100), and Epstein‐Barr virus‐In situ hybridization (EBV‐ISH) negative (X100).

As the use of CAR T‐cell therapy increases, the risk of secondary malignancies is becoming more recognized. Secondary T‐cell lymphoma is a rare and recently recognized complication of CAR T‐cell therapy. The possibility of malignant oncogenic transformation of transduced T‐cells was a concern surrounding the use of CAR T‐cell approaches. In the current case, there is no evidence of viral vector integration, and the development of T‐cell lymphoma 1 month after CAR T‐cell infusion raised the possibility of premalignant T‐cell clone evolution to lymphoma after CAR T‐cell therapy due to immune dysregulations. A recent review of second tumors after CAR T‐cell therapy noted these tumors are an emerging concern and found no evidence of oncogenic retroviral integration [1]. With the rising use of CAR T‐cell therapy, it is crucial for clinicians to recognize this emerging entity as a known risk. Overall, the low incidence of secondary T‐cell lymphomas reassures the safety of commercially available CAR T‐cell products.

## AUTHOR CONTRIBUTIONS

**Mingyi Chen**: Writing, Conceptualization and Final approval. **Joshua Mehr**: Writing and final approval.

## CONFLICT OF INTEREST STATEMENT

The authors declare no conflict of interest

## FUNDING INFORMATION

The authors received no specific funding for this work.

## ETHICS STATEMENT

Ethics and integrity standards were appropriately followed.

## PATIENT CONSENT STATEMENT

Patient consent is waived.

## CLINICAL TRIAL REGISTRATION

The authors have confirmed clinical trial registration is not needed for this submission.

## Data Availability

The data that support the findings of this study are available from the corresponding author upon reasonable request.
